# Photorespiration: origins and metabolic integration in interacting compartments

**DOI:** 10.1093/jxb/erw178

**Published:** 2016-05-13

**Authors:** Martin Hagemann, Andreas PM Weber, Marion Eisenhut

**Affiliations:** Universität Rostock; Heinrich-Heine-Universität, Düsseldorf; Heinrich-Heine-Universität, Düsseldorf

This special issue on photorespiration focuses on recent advances in this topic. The majority of the papers summarizes and extends contributions given at the 2nd workshop, ‘Photorespiration–key to better crops’, held in Warnemuende, Germany in June 2015. This was organized by the DFG (German Research Foundation)-supported research network, ‘Photorespiration: origins and metabolic integration in interacting compartments’ (FOR 1186–Promics).

The term photorespiration (PR) describes a light-induced biochemical process that converts 2-phosphoglycolate (2PG) into 3-phosphoglycerate (3PGA) and is accompanied by O_2_ uptake and CO_2_ release. It is closely associated with photosynthetic CO_2_ assimilation and represents one of the major highways of carbon metabolism in most plants. By mass flow, surpassed only by photosynthesis, PR actually constitutes the second most important process in the land-based biosphere. Plants using the most widespread C_3_ type of photosynthesis for CO_2_ assimilation display particularly massive photorespiratory CO_2_ production. PR is initiated by competition of O_2_ with CO_2_ at the active site of the universal carboxylating enzyme Ribulose 1,5-bisphosphate Carboxylase/Oxygenase (Rubisco) (Smith, 1976), which produces large amounts of 2PG during the day. Hence, PR essentially acts as a salvage or metabolic repair process that converts the toxic by-product 2PG into the useful Calvin–Benson cycle intermediate 3PGA. It is supposedly the most important ancient ancillary metabolic process that enables plants to thrive in an O_2_-containing atmosphere (Osmond, 1981). To convert 2PG into 3PGA, the concerted action of many plastidial, peroxisomal, mitochondrial, and also cytosolic enzymes is necessary, which makes this pathway the most prominent example of subcellular metabolic integration in higher plants.

However, PR also leads to the loss of a considerable fraction of freshly assimilated C and N as photorespiratory CO_2_ and NH_3_. Quantitatively, PR can decrease photosynthesis by up to 30% under current atmospheric concentrations of CO_2_ and O_2_ and even more at elevated temperature (e.g. Sharkey, 1988; Zhu *et al.*, 2004). This substantial decrease of net photosynthesis led to the somewhat misleading view of PR as a ‘wasteful’ process limiting photosynthetic productivity in C_3_ plants (e.g. Garrett, 1978; Siedow and Day, 2001). However, genetic analysis showed that PR is essential for all organisms performing oxygenic photosynthesis, since mutations of genes encoding for key photorespiratory enzymes always resulted in the photorespiratory phenotype and frequently in lethality (Somerville, 2001), i.e. corresponding mutants of cyanobacteria, red algae (Rademacher *et al*., this issue), chlorophytes, C_3_ and C_4_ plants were not viable in ambient air and could only be rescued under artificially enhanced CO_2_/O_2_ ratios (reviewed in Bauwe *et al.*, 2010). Nevertheless, due to the large CO_2_ and energy losses, PR is seen as a promising target in breeding more productive crops (Ort *et al.*, 2015). For example, attempts have been initiated to introduce photorespiratory bypasses to make the process less energy demanding or to reduce the CO_2_ release (reviewed in Peterhänsel *et al.*, 2013). Interestingly, it has recently been reported that, instead of decreasing PR an increased PR flux capacity resulted in enhanced growth of *Arabidopsis thaliana* in ambient air. The independent over-expression of two different proteins of the mitochondrial glycine cleavage system increased photorespiratory carbon flow and also improved Calvin–Benson cycle activity, leading to higher photosynthetic activity and higher biomass production (Timm *et al.*, 2012a, 2015).

Following the discovery between 1960 and 1980 of many essentials of the photorespiratory cycle (Tolbert, 1971; Ogren, 2003), research interest in this topic declined. However, the recognition of PR as a main crop-breeding target and the development of new tools for plant research resulted in a revitalization of PR research over the last 15 years. Meanwhile most contributing enzymes and genes have been identified at a molecular level and a set of corresponding mutants was generated to investigate the metabolic interaction of PR with the metabolism of the entire plant (e.g. Boldt *et al.*, 2005; Schwarte and Bauwe, 2007; Timm *et al.*, 2008, 2012b). For example, these results showed the close interaction of PR with plant C1-metabolism and with other metabolic pathways (Engel *et al.*, 2007; Ewald *et al.*, 2007). Advanced metabolomic and transcriptomic approaches allowed a system-wide characterization of PR in the wild type and mutants of Arabidopsis (e.g. Fernie *et al.*, 2013). The first candidates for photorespiratory transporters connecting the different interacting compartments such as the chloroplastidial glycerate/glycolate exchanger were identified by bioinformatics tools and then biochemically verified (Bordych *et al.*, 2013; Pick *et al.*, 2013). Research on C_4_ plants not only showed that PR is essential for these plants (Zelitch *et al.*, 2009), but also provided increasing evidence for the important role of PR in the evolution of C_4_ photosynthesis (Sage, 2004). The different location of photorespiratory enzymes in so-called C_3_–C_4_ intermediate plants generated/established an ancient CO_2_ pump based on the transport of glycine and serine, which is also called C_2_ photosynthesis, as the precursor for the final dicarboxyacid-based C_4_ cycle (e.g. Mallmann *et al.*, 2014). Last but not least, the identification of functional photorespiratory processes in cyanobacteria resulted in the view that photorespiration is an ancient process which coevolved with oxygenic photosynthesis (Eisenhut *et al.*, 2006, 2008; Kern *et al.*, 2011, 2013; Bauwe *et al.*, 2012; Hagemann *et al.*, 2013).

The present special issue reports on different aspects of actual PR research. It comprises one insight paper, eight review papers, three opinion papers, and nine original research papers.

The insight paper and three reviews discuss the current view on PR and its evolution. Sage (this issue) summarized the stepwise development of C_4_ photosynthesis from C_3_ photosynthesis, whereby the localization of photorespiratory enzymes and metabolic fluxes between bundle sheath and mesophyll played a crucial role. Bräutigam and Gowik (this issue) highlight the important role of PR in the evolution of C_4_ photosynthesis via intermediary stages, in which the capacity for PR is lost from leaf mesophyll cells and relocated to the bundle sheath cells. As shown by Döring *et al.* (this issue), in fully evolved C_4_ plants such as sorghum, the majority of genes encoding components of PR are also expressed preferentially in bundle sheath cells. Khoshravesh *et al*. (this issue) highlight the importance of organelle positioning in bundle sheath cells and the relocation of photorespiratory activity to this tissue during the evolution of C_4_ photosynthesis in grasses. Hagemann *et al*. (this issue) review the current position on the continuous coevolution of photosynthesis and PR. The evolution of all photorespiratory enzymes was elucidated and it was revealed that the present-day plant photorespiratory enzymes originated from archaeal, bacterial, and cyanobacterial sources, which served as eukaryotic host cell (Archaea), and mitochondrial (proteobacteria) or plastdial (cyanobacteria) endosymbionts, respectively. Moreover, calculating in terms of the geological era, ancient CO_2_/O_2_ ratios indicated that photorespiratory metabolism existed from the invention of oxygenic photosynthesis and remained necessary in cells evolving different types of carbon-concentrating mechanisms (CCM).

Another set of contributions deals with the intertwining of the photorespiratory pathway with the central metabolism and its significance for engineering plant productivity. For example, Fromm *et al*. (this issue), by using mutants that lack the activity of mitochondrial NADH dehydrogenase, analysed the role of the mitochondrial electron transport chain in photosynthesis. Using transcriptomic analysis of *Lotus japonicus* wild type and GS2 mutant plants on a range of different nitrogen concentrations and at ambient and elevated CO_2_, Pérez-Delgado *et al.* (this issue) show that primary nitrogen assimilation and PR are transcriptionally co-regulated. They also identify candidate transcription factors mediating this co-ordinated response. Betti *et al.* (this issue) summarize recent advances obtained in photorespiratory engineering and discuss the potential of realizing gains in crop productivity through manipulation of the photorespiratory pathway. Nunes-Nesi *et al.* (this issue) explore natural genetic variation in yield and photosynthetic capacity and point to the fact that photorespiratory capacity, in part, is genetically determined. Obata *et al.* (this issue) review the tight integration of PR with other metabolic pathways which reach beyond the recycling of carbon from the Calvin–Benson cycle, a topic that is further extended by Hodges *et al.* (this issue). Timm *et al.* (this issue) focus on the still limited understanding of regulatory interactions between plant PR and photosynthesis and discuss its critical impact for successfully engineering photosynthesis. Montgomery *et al.* (this issue) also explore the regulatory effect of light reactions on PR in their opinion paper. They employ the framework of modularity in the cyanobacterium *Fremyella diplosiphon* and suggest a highly controlled interplay among light reactions, PR, and CCM. The opinion paper by Orf *et al.* (this issue) also centres on cyanobacteria. According to their comparative meta-analysis of cyanobacterial and plant metabolite profiles, the authors propose that cyanobacteria can serve as a much simpler surrogate to study the complex, highly compartmentalized, plant PR metabolism.

A deeper understanding of PR requires technology to determine rates of PR and photosynthesis accurately and this is reviewed by Hanson *et al.* (this issue). Alonso-Cantabrana and von Caemmerer (this issue) report on using carbon isotope discrimination as a tool to quantify C_4_-like photosynthesis in C_3_–C_4_ intermediate species. Labelling with the stable carbon isotope ^13^C also revealed a strong effect of reduced mitochondrial malate dehydrogenase activity on PR (Lindén *et al.*, this issue). Sharwood *et al.* (this issue) emphasize the importance of standardized and validated protocols for quantifying carbon fixation capacity in plants with differing carbon assimilation strategies, with particular emphasis on quantifying Rubisco activity.

Four papers deal with the specific role of the central enzyme, glycolate oxidase. The biochemistry of this peroxisomal enzyme converting glycolate into glyoxylate is reviewed by Hodges *et al*. (this issue). Dellero *et al*. report on the impact of reduced photorespiratory glycolate oxidase activity on leaf metabolism in Arabidopsis (Dellero *et al.*, a, this issue) and review recent advances in the understanding of glyoxylate metabolism in different plant organs (Dellero *et al.*, b, this issue). Knocking out glycolate oxidase of *Cyanidioschyzon merolae* resulted in the first mutant with a photorespiratory phenotype among red algae (Rademacher *et al*., this issue). This finding revealed that the plant-type photorespiratory cycle using a peroxisomal glycolate oxidase evolved before the split of red and green algae, and it represents a further example that organisms, though carrying a CCM, also depend on functional PR.

## Concluding remarks

The past decades of research on the process of PR have revealed that this pathway is an indispensable companion to oxygenic photosynthesis and includes photosynthetic organisms that feature highly efficient CCMs such as cyanobacteria, many algae, and C_4_ plants. Not only is it essential to support photosynthetic carbon assimilation, it is also heavily intertwined with other metabolic pathways and is the driving force for the evolution of C_4_ photosynthesis (Heckmann *et al.*, 2013), the most efficient mode of photosynthetic carbon assimilation in the angiosperms. In extant oxygenic photosynthetic organisms, the only means to reduce the rate of PR and hence enhance photosynthetic efficiency is to increase the concentration of CO_2_ at the site of Rubisco. Therefore, future research aimed at increasing the efficiency of photosynthesis in crop plants might want to focus on this aspect. In the short term, perhaps the most promising path towards increased crop efficiency might be founded on a deeper understanding of natural variation in photosynthetic capacity to unravel those genes that determine source strength. Here, a better understanding of PR regulation and its integration into the cellular metabolism via yet unidentified transporters (e.g. exchanging glycine and serine between mitochondria and peroxisomes) would be an important future aim. However, in the long-term, it is also envisaged that synthetic pathways for carbon assimilation (i.e. pathways not existing in nature), that are not affected by oxygen, might become a reality (Bar-Even *et al.*, 2010; Ort *et al.*, 2015), thereby enabling hitherto unimaginable gains in productivity.

## References

[CIT0001] Alonso-Cantabrana H von Caemmerer S 2016 Carbon isotope discrimination as a diagnostic tool for C_4_ photosynthesis in C_3_–C_4_ intermediate species. Journal of Experimental Botany 67, 3109–3121. 10.1093/jxb/erv555 PMC4867892 26862154

[CIT0002] Bar-Even A Noor E Lewis NE Milo R 2010 Design and analysis of synthetic carbon fixation pathways. Proceedings of the National Academy of Sciences, USA 107, 8889–8894. 10.1073/pnas.0907176107 PMC2889323 20410460

[CIT0003] Bauwe H Hagemann M Fernie AR 2010 Photorespiration: players, partners and origin. Trends in Plant Science 15, 330–336. 20403720 10.1016/j.tplants.2010.03.006

[CIT0004] Bauwe H Hagemann M Kern R Timm S 2012 Photorespiration has a dual origin and manifold links to central metabolism. Current Opinion in Plant Biology 15, 269–275. 22284850 10.1016/j.pbi.2012.01.008

[CIT0005] Betti M Bauwe H Busch FA 2016 Manipulating photorespiration to increase plant productivity: recent advances and perspectives for crop improvement. Journal of Experimental Botany 67, 2977–2988. 10.1093/jxb/erw076 26951371

[CIT0006] Boldt R Edner C Kolukisaoglu Ü Hagemann M Weckwerth W Wienkoop S Morgenthal K Bauwe H 2005 d-Glycerate 3-kinase, the last unknown enzyme in the photorespiratory cycle in Arabidopsis, belongs to a novel kinase family. The Plant Cell 17, 2413–2420. 15980259 10.1105/tpc.105.033993 PMC1182498

[CIT0007] Bordych C Eisenhut M Pick TR Kuelahoglu C Weber APM 2013 Co-expression analysis as tool for the discovery of transport proteins in photorespiration. Plant Biology (Stuttgart) 15, 686–693. 10.1111/plb.12027 23590453

[CIT0008] Bräutigam A Gowik U 2016 Photorespiration connects C3 and C4 photosynthesis. Journal of Experimental Botany 67, 2953–2962. 10.1093/jxb/erw056 26912798

[CIT0009] Dellero Y Jossier M Glab N Oury C Tcherkez G Hodges M 2016a. Decreased glycolate oxidase activity leads to altered carbon allocation and leaf senescence after a transfer from high CO_2_ to ambient air in *Arabidopsis thaliana* . Journal of Experimental Botany 67, 3149–3163. 10.1093/jxb/erw054 26896850

[CIT0010] Dellero Y Jossier M Schmitz J Maurino VG Hodges M 2016b. Photorespiratory glycolate-glyoxylate metabolism. Journal of Experimental Botany 67, 3041–3052. 10.1093/jxb/erw090 26994478

[CIT0011] Döring F Streubel M Bräutigam A Gowik U 2016 Most photorespiratory genes are preferentially expressed in the bundle sheath cells of the C_4_ grass *Sorghum bicolor* . Journal of Experimental Botany 67, 3053–3064. 10.1093/jxb/erw041 PMC4867894 26976818

[CIT0012] Eisenhut M Kahlon S Hasse D Ewald R Lieman-Hurwitz J Ogawa T Ruth W Bauwe H Kaplan A Hagemann M 2006 The plant-like C2 glycolate cycle and the bacterial-like glycerate pathway cooperate in phosphoglycolate metabolism in cyanobacteria. Plant Physiology 142, 333–342. 16877700 10.1104/pp.106.082982 PMC1557606

[CIT0013] Eisenhut M Ruth W Haimovich M Bauwe H Kaplan A Hagemann M 2008 The photorespiratory glycolate metabolism is essential for cyanobacteria and might have been conveyed endosymbiontically to plants. Proceedings of the National Academy of Sciences, USA 105, 17199–17204. 10.1073/pnas.0807043105 PMC2579401 18957552

[CIT0014] Engel N van den Daele K Kolukisaoglu Ü Morgenthal K Weckwerth W Pärnik T Keerberg O Bauwe H 2007 Deletion of glycine decarboxylase in Arabidopsis is lethal under non-photorespiratory conditions. Plant Physiology 144, 1328–1335. 17496108 10.1104/pp.107.099317 PMC1914133

[CIT0015] Ewald R Kolukisaoglu Ü Bauwe U Mikkat S Bauwe H 2007 Mitochondrial protein lipoylation does not exclusively depend on the mtKAS pathway of de-novo fatty acid synthesis in Arabidopsis. Plant Physiology 145, 41–48. 17616510 10.1104/pp.107.104000 PMC1976585

[CIT0016] Fernie AR Bauwe H Eisenhut M 2013 Perspectives on plant photorespiratory metabolism. Plant Biology (Stuttgart) 15, 748–753. 10.1111/j.1438-8677.2012.00693.x 23231538

[CIT0017] Fromm S Senkler S Eubel H Peterhänsel C Braun HP 2016 Life without complex I: proteome analyses of an Arabidopsis mutant lacking the mitochondrial NADH dehydrogenase complex. Journal of Experimental Botany 67, 3079–3093. 10.1093/jxb/erw165 PMC4867900 27122571

[CIT0018] Garrett MK 1978 Control of photorespiration at RuBP carboxylase/oxygenase level in ryegrass cultivars. Nature 274, 913–915.

[CIT0019] Hagemann M Fernie AR Espie GS Kern R Eisenhut M Reumann S Bauwe H Weber APM 2013 Evolution of the biochemistry of the photorespiratory C2 cycle. Plant Biology (Stuttgart) 15, 639–647. 10.1111/j.1438-8677.2012.00677.x 23198988

[CIT0020] Hagemann M Kern R Maurino VG Hanson DT Weber AP Sage RF Bauwe H 2016 Evolution of photorespiration from cyanobacteria to land plants, considering protein phylogenies and acquisition of carbon concentrating mechanisms. Journal of Experimental Botany 67, 2963–2976. 10.1093/jxb/erw063 26931168

[CIT0021] Hanson ST Stutz SS Boyer JS 2016 Why small fluxes matter: the case and approaches for improving measurements of photosynthesis and (photo)respiration. Journal of Experimental Botany 67, 3027–3039. 10.1093/jxb/erw139 PMC4867897 27099373

[CIT0022] Heckmann D Schulze S Denton A Gowik U Westhoff P Weber APM Lercher M 2013 Predicting C_4_ photosynthesis evolution: modular, individually adaptive steps on a Mount Fuji Fitness Landscape. Cell 153, 1579–1588. 23791184 10.1016/j.cell.2013.04.058

[CIT0023] Hodges M Dellero Y Keech O Betti M Raghavendra AS Sage R Zhu X Allen DK Weber APM 2016 Perspectives for a better understanding of the metabolic integration of photorespiration within a complex plant primary metabolism network. Journal of Experimental Botany 67, 3015–3026. 10.1093/jxb/erw145 27053720

[CIT0024] Kern R Bauwe H Hagemann M 2011 Evolution of enzymes involved in the photorespiratory 2-phosphoglycolate cycle from cyanobacteria via algae toward plants. Photosynthesis Research 109, 103–114. 21222161 10.1007/s11120-010-9615-z

[CIT0025] Kern R Eisenhut M Bauwe H Weber APM Hagemann M 2013 Does the Cyanophora paradoxa genome revise our view on the evolution of photorespiratory enzymes? Plant Biology (Stuttgart) 15, 759–768. 10.1111/plb.12003 23551942

[CIT0026] Khoshravesh R Stinson C Stata M Busch F Sage RF Ludwig M Sage TL 2016 Facilitating C_2_ photosynthesis in grasses: centripetal organelle enrichment and cell-specific GDC expression in bundle sheath cells. Journal of Experimental Botany 67, 3065–3078. 10.1093/jxb/erw150 PMC4867898 27073202

[CIT0027] Lindén P Keech O Stenlund H Gardeström P Moritz T 2016 Reduced mitochondrial malate dehydrogenase activity has a strong effect on photorespiratory metabolism as revealed by ^13^C-labelling. Journal of Experimental Botany 67, 3123–3135. 10.1093/jxb/erw030 PMC4867893 26889011

[CIT0028] Mallmann J Heckmann D Bräutigam A Lercher MJ Weber AP Westhoff P Gowik U 2014 The role of photorespiration during the evolution of C_4_ photosynthesis in the genus *Flaveria* . Elife 3, e02478. 24935935 10.7554/eLife.02478 PMC4103682

[CIT0029] Montgomery BL Lechno-Yossef S Kerfeld CA 2016 Interrelated modules in cyanobacterial photosynthesis: the carbon concentrating mechanism, photorespiration and light perception. Journal of Experimental Botany 67, 2931–2940. 10.1093/jxb/erw162 27117337

[CIT0030] Nunes-Nesi A Nascimento VL Silva FMO Zsogon A Araújo WL Sulpice R 2016 Natural genetic variation for morphological and molecular determinants of plant growth and yield. Journal of Experimental Botany 67, 2989–3001. 10.1093/jxb/erw124 27012286

[CIT0031] Obata T Florian A Timm S Bauwe H Fernie AR 2016 On the metabolic interaction of (photo)respiration. Journal of Experimental Botany 67, 3003–3014. 10.1093/jxb/erw128 27029352

[CIT0032] Ogren WL 2003 Affixing the O to Rubisco: discovering the source of photorespiratory glycolate and its regulation. Photosynthesis Research 76, 53–63. 16228565 10.1023/A:1024913925002

[CIT0033] Orf I Timm S Bauwe H Fernie AR Hagemann M Kopka J Nikoloski Z 2016 Can cyanobacteria serve as a model of plant photorespiration? A comparative meta-analysis of metabolite profiles. Journal of Experimental Botany 67, 2941–2952. 10.1093/jxb/erw068 26969741

[CIT0034] Ort DR Merchant SS Alric J 2015 Redesigning photosynthesis to sustainably meet global food and bioenergy demand. Proceedings of the National Academy of Sciences, USA 112, 8529–8536. 10.1073/pnas.1424031112 PMC4507207 26124102

[CIT0035] Osmond CB 1981 Photorespiration and photoinhibition. Some implications for the energetics of photosynthesis. Biochimica et Biophysica Acta 639, 77–98.

[CIT0036] Pérez-Delgado CM Moyano TC García-Calderón M Canales J Gutiérrez RA Márquez AJ Betti M 2016 Use of transcriptomics and co-expression networks to analyse the interconnections between nitrogen assimilation and photorespiratory metabolism. Journal of Experimental Botany 67, 3095–3108. 10.1093/jxb/erw170 PMC4867901 27117340

[CIT0037] Peterhänsel C Krause K Braun HP Espie GS Fernie AR Hanson DT Keech O Maurino VG Mielewczik M Sage RF 2013 Engineering photorespiration: current state and future possibilities. Plant Biology (Stuttgart) 15, 754–758. 10.1111/j.1438-8677.2012.00681.x 23121076

[CIT0038] Pick TR Bräutigam A Schulz MA Obata T Fernie AR Weber APM 2013 PLGG1, a plastidic glycolate glycerate transporter, is required for photorespiration and defines a unique class of metabolite transporters. Proceedings of the National Academy of Sciences, USA 110, 3185–3190. 10.1073/pnas.1215142110 PMC3581909 23382251

[CIT0039] Rademacher N Kern R Fujiwara T Mettler-Altmann T Miyagishima SY Hagemann M Eisenhut M Weber AP 2016 Photorespiratory glycolate oxidase is essential for the survival of the red alga *Cyanidioschyzon merolae* under ambient CO_2_ conditions. Journal of Experimental Botany 67, 3165–3175. 10.1093/jxb/erw118 PMC4867895 26994474

[CIT0040] Sage RF 2004 The evolution of C_4_ photosynthesis. New Phytologist 161, 341–370. 10.1111/j.1469-8137.2004.00974.x 33873498

[CIT0041] Sage RF 2016 Tracking the evolutionary rise of C_4_ metabolism. Journal of Experimental Botany 67, 2919-2922. 10.1093/jxb/erw137 PMC4867896 27085185

[CIT0042] Schwarte S Bauwe H 2007 Identification of the photorespiratory 2-phosphoglycolate phosphatase, PGLP1, in Arabidopsis. Plant Physiology 144, 1580–1586. 17478634 10.1104/pp.107.099192 PMC1914141

[CIT0043] Sharkey TD 1988 Estimating the rate of photorespiration in leaves. Physiologia Plantarum 73, 147–152.

[CIT0044] Sharwood RE Sonawane B Ghannoum O Whitney S 2016 Improved analysis of C_4_ and C_3_ photosynthesis via refined *in vitro* assays of their carbon fixation biochemistry. Journal of Experimental Botany 67, 3137–3148. 10.1093/jxb/erw154 PMC4867899 27122573

[CIT0045] Siedow JN Day DA 2001 Respiration and photorespiration. In: Buchanan BB Gruissem W Jones RL (eds). *Biochemistry and molecular biology of plants*.American Society of Plant Physiologists: Rockville, 676–728.

[CIT0046] Smith BN 1976 Evolution of C4 photosynthesis in response to changes in carbon and oxygen concentrations in the atmosphere through time. BioSystems 8, 24–32. 953157 10.1016/0303-2647(76)90004-6

[CIT0047] Somerville CR 2001 An early Arabidopsis demonstration. Resolving a few issues concerning photorespiration. Plant Physiology 125, 20–24. 11154287 10.1104/pp.125.1.20 PMC1539316

[CIT0048] Timm S Florian A Arrivault S Stitt M Fernie AR Bauwe H 2012 *a* Glycine decarboxylase controls photosynthesis and plant growth. FEBS Letters 586, 3692–3697. 22982108 10.1016/j.febslet.2012.08.027

[CIT0049] Timm S Florian A Fernie AR Bauwe H 2016 The regulatory interplay between photorespiration and photosynthesis. Journal of Experimental Botany 67, 2923–2929. 10.1093/jxb/erw083 26969745

[CIT0050] Timm S Mielewczik M Florian A Frankenbach S Dreissen A Hocken N Fernie AR Walter A Bauwe H 2012 *b* High-to-low CO_2_ acclimation reveals plasticity of the photorespiratory pathway and indicates regulatory links to cellular metabolism of Arabidopsis. PLoS One 7, e42809. 22912743 10.1371/journal.pone.0042809 PMC3422345

[CIT0051] Timm S Nunes-Nesi A Pärnik T Morgenthal K Wienkoop S Keerberg O Weckwerth W Kleczkowski LA Fernie AR Bauwe H 2008 A cytosolic pathway for the conversion of hydroxypyruvate to glycerate during photorespiration in Arabidopsis. The Plant Cell 20, 2848–2859. 18952776 10.1105/tpc.108.062265 PMC2590732

[CIT0052] Timm S Wittmiß M Gamlien S Ewald R Florian A Frank M Wirtz M Hell R Fernie AR Bauwe H 2015 Mitochondrial dihydrolipoyl dehydrogenase activity shapes photosynthesis and photorespiration of *Arabidopsis thaliana* . The Plant Cell 27, 1968–1984. 26116608 10.1105/tpc.15.00105 PMC4531348

[CIT0053] Tolbert NE 1971 Microbodies—peroxisomes and glyoxysomes. Annual Review of Plant Physiology 22, 45–74.

[CIT0054] Zelitch I Schultes NP Peterson RB Brown P Brutnell TP 2009 High glycolate oxidase activity is required for survival of maize in normal air. Plant Physiology 149, 195–204. 18805949 10.1104/pp.108.128439 PMC2613714

[CIT0055] Zhu XG Portis AR Long SP 2004 Would transformation of C_3_ crop plants with foreign Rubisco increase productivity? A computational analysis extrapolating from kinetic properties to canopy photosynthesis. Plant, Cell and Environment 27, 155–165.

